# Ramipril mitigates radiation-induced impairment of neurogenesis in the rat dentate gyrus

**DOI:** 10.1186/1748-717X-5-6

**Published:** 2010-02-01

**Authors:** Kenneth A Jenrow, Stephen L Brown, Jianguo Liu, Andrew Kolozsvary, Karen Lapanowski, Jae Ho Kim

**Affiliations:** 1 Henry Ford Hospital, Department of Neurosurgery, 3074 E&R Building, 2799 W Grand Boulevard, Detroit, Michigan 48202, USA; 2 Henry Ford Hospital, Department of Radiation Oncology, HFH-M2, 2799 W Grand Boulevard, Detroit, Michigan 48202, USA

## Abstract

**Background:**

Sublethal doses of whole brain irradiation (WBI) are commonly administered therapeutically and frequently result in late delayed radiation injuries, manifesting as severe and irreversible cognitive impairment. Neural progenitors within the subgranular zone (SGZ) of the dentate gyrus are among the most radiosensitive cell types in the adult brain and are known to participate in hippocampal plasticity and normal cognitive function. These progenitors and the specialized SZG microenvironment required for neuronal differentiation are the source of neurogenic potential in the adult dentate gyrus, and provide a continuous supply of immature neurons which may then migrate into the adjacent granule cell layer to become mature granule cell neurons. The extreme radiosensitivity of these progenitors and the SGZ microenvironment suggests the hippocampus as a prime target for radiation-induced cognitive impairment. The brain renin-angiotensin system (RAS) has previously been implicated as a potent modulator of neurogenesis within the SGZ and selective RAS inhibitors have been implicated as mitigators of radiation brain injury. Here we investigate the angiotensin converting enzyme (ACE) inhibitor, ramipril, as a mitigator of radiation injury in this context.

**Methods:**

Adult male Fisher 344 rats received WBI at doses of 10 Gy and 15 Gy. Ramipril was administered beginning 24 hours post-WBI and maintained continuously for 12 weeks.

**Results:**

Ramipril produced small but significant reductions in the deleterious effects of radiation on progenitor proliferation and neuronal differentiation in the rat dentate gyrus following 10 Gy-WBI, but was not effective following 15 Gy-WBI. Ramipril also reduced the basal rate of neurogenesis within the SGZ in unirradiated control rats.

**Conclusions:**

Our results indicate that chronic ACE inhibition with ramipril, initiated 24 hours post-irradiation, may reduce apoptosis among SGZ progenitors and/or inflammatory disruption of neurogenic signaling within SGZ microenvironment, and suggest that angiotensin II may participate in maintaining the basal rate of granule cell neurogenesis.

## Background

Sublethal doses of whole brain irradiation (WBI) are commonly administered therapeutically (cranial radiation), and might also be administered inadvertently in the event of a nuclear accident or radiological attack [1-3]. Clinical data derived from patients receiving cranial radiation suggest that long term survivors of such exposures are at risk for developing late delayed effects manifesting as chronic and irreversible cognitive impairment and dementia [3]. These late delayed effects are routinely observed following WBI doses substantially below thresholds for vasculopathy or demyelination, but sufficient to impair granule cell neurogenesis within the hippocampus along with electrophysiological and behavioral measures of hippocampal plasticity [4-12]. These observations suggest that impaired neurogenesis and plasticity within the hippocampus may contribute to cognitive impairment in humans exposed to WBI, and that mitigating radiation damage to these progenitors and/or preserving their neurogenic potential might be a successful strategy for reducing the development of these late delayed effects.

The learning and memory functions of the hippocampus have been associated with a coordinated neurogenic response that occurs within the subgranular zone (SGZ) of the dentate gyrus, one of only two regions in the adult brain (the other being the subventricular zone) where the capacity for neurogenesis is retained throughout life [4,13,14]. The unique microenvironment within the SGZ induces vascular adventitial stem cells to differentiate into rapidly dividing progenitors, which are typically found in discrete clusters surrounding their source microvessels [4,5,15]. Signaling within the SGZ microenvironments defined by these clusters is required for neuronal differentiation among the progenitors and coordinates the rate of neurogenesis with the demands of hippocampally-mediated learning and memory processes [13,14]. Immature neurons may then migrate away from these clusters into the adjacent granule cell layer (GCL) where they may gradually mature to assume the morphological and functional characteristics of granule cell neurons [9,16]. The proportion of these neurons that survive to become mature granule cell neurons is generally small but can be increased by behavioral activity, including physical exercise, environmental enrichment, and spatial learning [16]. During their maturation, which requires approximately 65 days, these new neurons are hyperexcitable and possess an enhanced potential for synaptic plasticity [11,16].

Ablating neurogenesis within the dentate gyrus impairs hippocampal plasticity and performance in spatial learning tasks, and the severity of this impairment is proportional to the extent of damage specific to the granule cell progenitor population [5,10,11,14,17]. Radiation dose-dependent decreases in granule cell neurogenesis are well established following WBI and result from the loss of neural progenitors, via apoptosis and mitotic catastrophe, and a disruption of neurogenic signaling, via the dispersion of progenitor clusters within the SGZ. These pathologies are inversely correlated with radiation dose-dependent increases in activated microglia within the dentate gyrus [18]. In vitro studies have revealed that activated microglia contribute to the disruption of neurogenesis in this context by releasing interleukin-6 (IL-6), interleukin-1β (IL-1β), and tumor necrosis factor-α (TNF-α), proinflammatory cytokines which induce a nonspecific decrease in cell survival as well as a selective decrease in neuronal differentiation among progenitor cells [9,18]. Administering the anti-inflammatory drug, indomethacin, or the PPARα agonist, fenofibrate, prior to irradiation partially prevents microglial activation and the decrease in neurogenesis post-irradiation. Thus reducing inflammation within the dentate gyrus post-irradiation might similarly reduce the deleterious effects of WBI on granule cell neurogenesis [9,19].

Inhibiting the renin-angiotensin system (RAS) has proven to be one of the more successful strategies for mitigating the development of late delayed effects following WBI doses above the necrotic threshold [20-22]. Though traditionally viewed as a blood-borne hormonal system, a number of intraorgan RASs have also been identified [23]. In the brain, RAS components are localized in both neuronal and glial cells which release renin and angiotensinogen. These peptides interact to produce angiotensin I, a biologically inactive decapeptide that is subsequently cleaved by angiotensin I converting enzyme (ACE) to produce the effector octapeptide angiotensin II (Ang II). Ang II is a potent vasopressor that exerts its effects by binding to G-protein receptors, AT1R and AT2R, which are broadly distributed in the brain and particularly dense in the hippocampus [23]. After tissue injury, AT2Rs are upregulated and Ang II, in combination with other cytokines and growth factors, produces pro-apoptotic, pro-inflammatory, and pro-oxidant effects that may participate in the development of long term tissue injury [24-31]. RAS inhibitors act either by inhibiting ACE (ACEi), thereby blocking the conversion of Ang I to Ang II, or by antagonizing the binding of Ang II with either of its receptor subtypes [23]. Using the ACEi, ramipril, we previously reported that chronic ramipril administration, initiated two weeks after 30 Gy focal irradiation of the rat brain, significantly mitigates the development of white matter necrosis in the optic tract [20-22]. Here we report that ramipril, administered 24 hours post-WBI and maintained daily for 12 weeks, reduces the deleterious effects of 10 Gy-WBI, but not 15 Gy-WBI, on neurogenesis in the rat dentate gyrus. Ramipril also reduces the basal rate of granule cell neurogenesis in unirradiated control rats, suggesting that Ang II participates in maintaining granule cell neurogenesis.

## Methods

Adult male Fischer 344 rats (Charles River Breeding Lab, Wilmington, MA) weighing between 200 and 240 g were used in all experiments. All animal procedures were performed in AAALAC accredited facilities, following institutionally approved protocols, in accordance with published recommendations for the proper care and use of laboratory animals. Food and water were provided ad libitum and consumption rates monitored daily. A total of 33 rats were randomly assigned to Radiation Treatment groups receiving WBI at prescribed doses of 0 (Control, n = 9), 10 Gy (n = 16), and 15 Gy (n = 8). Each of these Radiation Treatment groups was subdivided into drug treatment groups receiving either ramipril (RAM) or vehicle only (RAD), administered according to the same schedule. Ramipril therapy was initiated 24 hours post-WBI, and maintained continuously until sacrifice at 12 weeks post-WBI.

Rats were irradiated using a dedicated self-shielded 5000Ci ^137^Cs irradiator (Mark I, Model 68, J.L. Shepherd and Associates, San Fernando, CA), with a primary collimator used to create a 2 cm × 30 cm rectangular dose field. Rats were anesthetized using ketamine (80 mg/kg) and xylazine (8 mg/kg) and positioned horizontally with their heads at the midpoint of this field (centered 15 cm above the base and 6 cm forward of the collimator face) to optimize uniformity of the dose distribution. Secondary lead shielding (1 cm thick) was used to limit radiation exposure to structures outside the brain including the jaw, pharynx, nose, and eyes. The head was oriented such that the radioactive source was lateral to the midline, with the 2 cm dose field dimension encompassing the anterior-posterior extent of the brain. To compensate for the affects of tissue attenuation, the prescribed radiation dose was administered bilaterally in two consecutive half-dose fractions. The measured dose rate at the time of irradiation was approximately 3.2 Gy/minute.

Ramipril treated rats (RAM subgroups) received a daily ramipril dose of approximately 1.5 mg/kg delivered by addition of the compound to their drinking water, whereas untreated rats (RAD subgroups) received bottle changes according the same schedule. The ramipril concentration in the drinking water was based on animal weight and the average ad libitum water consumption rate of approximately 20 ml/day measured among our experimental animals. Ramipril is an ester-containing prodrug that is rapidly absorbed after oral intake and its absorption is not affected by food. Upon absorption, ramipril is metabolized by the liver and converted into its active form, ramiprilat, a potent ACE inhibitor. The bioavailability of ramipril is highly predictable and the stability of the drug in drinking water is superior to other ACE inhibitors. The drug also has the demonstrated ability to cross the blood-brain barrier, unlike many other clinically available ACE inhibitors [32,33].

Rats were sacrificed under deep pentobarbital anesthesia (80 mg/kg) by transcardial perfusion with saline (300 ml) followed by 10% neutral buffered formalin (300 ml). Brains were removed and post-fixed overnight at 4°C in 10% neutral buffered formalin, coronally sectioned into 2 mm blocks, and processed for paraffin embedding. Groups of four serial sections (7 μm thickness) were cut at 50 μm intervals along the rostral caudal axis of the hippocampus. Within each of these groups, one section was stained with hematoxylin-eosin (H&E) for routine histological assessment, whereas the three remaining sections were stained immunohistochemically using antibodies for Ki-67 (1:100, 60 min, Thermo Fisher Scientific, Fremont, CA,), a selective marker of cellular proliferation; doublecortin (DCX; 1:100, overnight at 4°C, Santa Cruz Biotechnology, Santa Cruz, CA), a selective marker of immature neurons; and CD68 (1:200, 30 min, AbD Serotec, Oxford, UK) a selective marker of activated microglia. Immunohistochemically stained sections were counter-stained using either hematoxylin (CD68) or DAPI (Ki-67 and DCX), as appropriate. For immunohistochemical processing, sections were deparaffinized and rehydrated, boiled for 10 minutes in 10 mM citrate buffer, incubated with primary antibodies, and labeled with DAB (CD68; 4+ detection/Betazoid DAB: Biocare Medical) or Cy3 (Ki-67 and DCX; 1:250 Alexa 555 secondary, Invitrogen) per manufacturer's instructions.

**Figure 1:**
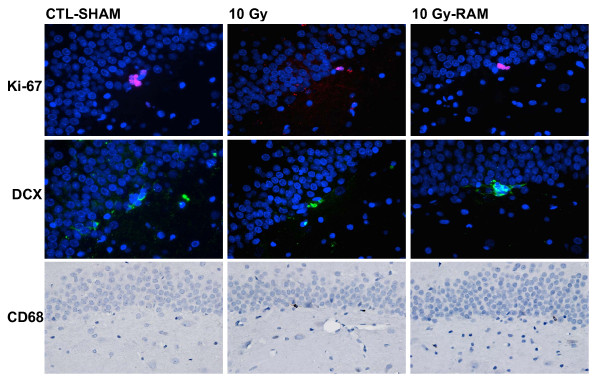
**Representative images of immunohistochemical staining for Ki-67^+ ^progenitors (red), DCX^+ ^immature neurons (green), and CD68^+ ^activated microglia (brown) in the SGZ and GCL, obtained at 400× from CLT-SHAM, 10 Gy, and 10 Gy-RAM group rats sacrificed at 12 weeks post-irradiation**. Note the robust progenitor and immature neuron production, and sparse activated microglia, in CTL-SHAM tissue. Progenitor and immature neuron production are severely impaired, and activated microglia increased, in 10 Gy tissues. Impaired progenitor and immature neuron production are subtly but significantly improved in 10 Gy-RAM tissue.

All analyses were performed by individuals naïve to the experimental conditions using previously established methods [6,8,34-36]. Cell counts were performed bilaterally and exhaustively at 400× within the designated fields. Counts of Ki-67^+ ^proliferating cells were performed within the SGZ (defined as the region extending 25 μm on either side of the border between the hilus and the GCL), and were restricted to cells with uniform cytoplasmic staining of a clearly demarcated spherical or elliptical structure containing a spherical DAPI-stained nucleus. Counts of DCX^+ ^immature neurons were performed within the GCL and SGZ, and were restricted to cells with uniform cytoplasmic staining of a clearly demarcated spherical or pyramidal structure (often with dendritic processes extending through the GCL toward the molecular layer) containing a spherical DAPI-stained nucleus. Counts of CD68^+ ^activated microglia were performed within the GCL and SGZ, and were restricted to cells with cytoplasmic staining of a small cell body and/or microglial processes associated with an elliptical nucleus. The length of the SGZ at the GCL-hilar boarder and the volume of the SGZ and GCL were calculated for each section from which counts were obtained. SGZ lengths were used to standardize Ki-67 and DCX counts as linear densities. The SGZ and GCL volumes were used to normalize the CD68 counts as volume densities [34,35]. (Fig. 1)

The average linear density of Ki-67^+ ^cells residing within the SGZ and inferior margin of the GCL was used as measure of granule cell progenitor proliferation near the time of sacrifice for each rat. The average linear density of DCX^+ ^cells residing within the SGZ and GCL was used as a measure of neurogenic potential near the time of sacrifice for each rat. The average volume density of CD68^+ ^cells residing within the SGZ and GCL was used as a measure of microglial activation for each rat. Group means and standard deviations were calculated from these data and, when necessary, log transformations were performed prior to analysis to adjust for unequal variances. These averages were statistically analyzed using analysis of variance (ANOVA) and Student's t to test whether: 1. Granule cell progenitor proliferation and/or neurogenesis were differentially affected in the Control group by RAM (relative to vehicle only); 2. Granule cell progenitor proliferation and/or neurogenesis were decreased in the 10 Gy- and 15 Gy-WBI Radiation Treatment groups relative to Control; 3. Decreases in granule cell progenitor proliferation and/or neurogenesis in the 10 Gy- and 15 Gy-WBI Radiation Treatment groups were reduced by RAM (relative to vehicle only); and 4. Microlglial activation was increased in the 10 Gy- and 15 Gy-WBI Radiation Treatment groups relative to Control and whether these increases were reduced by RAM (relative to vehicle only) [37].

## Results

**Figure 2:**
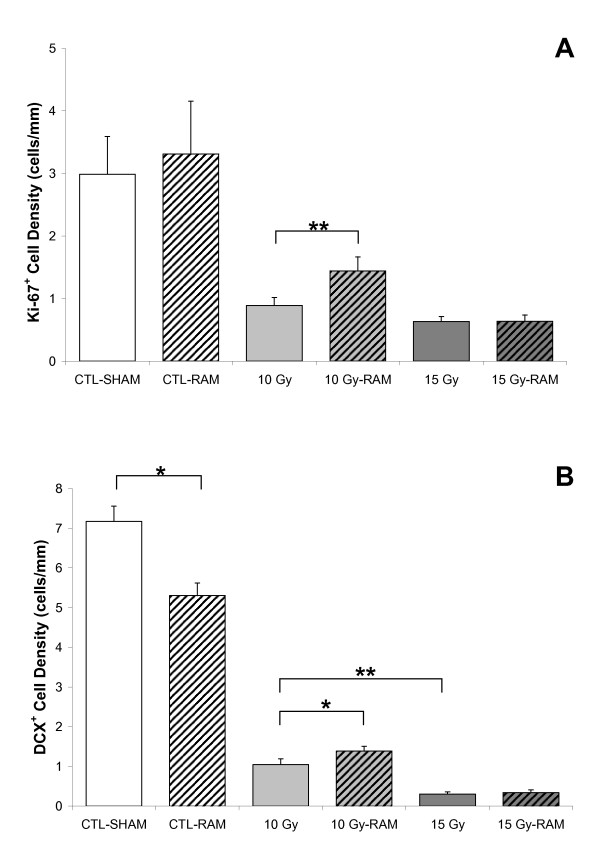
**Linear densities of Ki-67^+ ^progenitors and DCX^+ ^immature neurons within the SGZ at 12 weeks post-irradiation**. **A**. Relative to CTL-SHAM, Ki-67^+ ^progenitor proliferation is significantly (p < 0.001) reduced following 10 Gy- and 15 Gy-WBI. Ramipril mitigates the reduction in Ki-67^+ ^progenitors following 10 Gy-WBI (p < 0.05), but not following 15 Gy-WBI. **B**. Relative to CTL-SHAM, DCX^+ ^immature granule cell neurons are significantly (p < 0.001) and dose-dependently (p < 0.01) reduced following 10 Gy- and 15 Gy-WBI. Ramipril mitigates the reduction in neurogenesis following 10 Gy-WBI (p < 0.05), but not following 15 Gy-WBI. Error bars represent the standard error of the mean for each treatment group. (* = p < 0.05; ** = p < 0.01).

**Figure 3:**
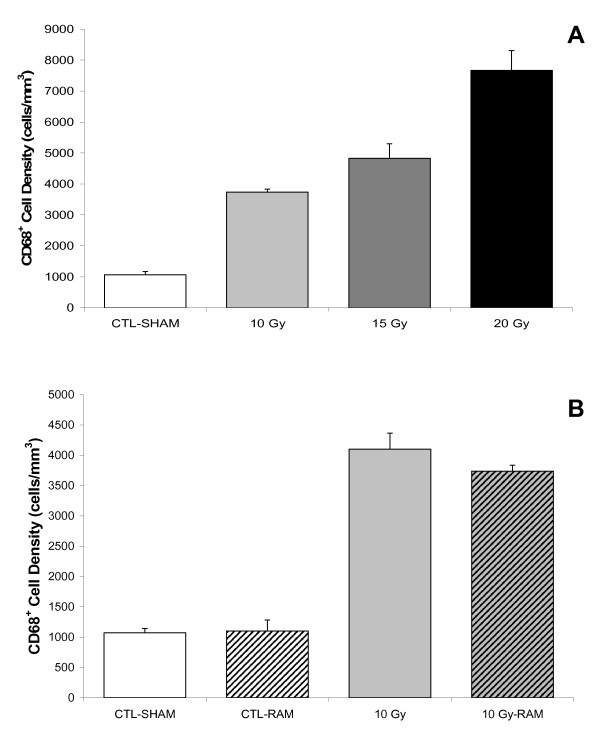
**Volume densities of activated microglia measured within the SGZ and GCL at 12 weeks post-irradiation**. **A**. Relative to CTL-SHAM, CD68^+^ activated microglia in the SGZ are significantly (p < 0.001) and dose-dependently increased following 10 Gy- and 15 Gy-WBI. **B**. CD68^+^ activated microglia are unaffected by ramipril in either the Control group (CTL-SHAM vs. CTL-AVS) or in the 10 Gy-WBR group (10 Gy vs. 10 Gy-AVS). Thus, the mitigating effects of ramipril on granule cell progenitor proliferation and neurogenesis following 10 Gy-WBI are not accompanied by a decrease in activated microglia. Error bars represent the standard error of the mean for each treatment group.

Food and water intake and body weight among our animals were not affected by either radiation or ramipril treatment during 12 weeks of monitoring post-irradiation. In the Control group (CTL-SHAM), progenitor proliferation (Ki-67) within the SGZ did not differ between the Vehicle and RAM treatment groups; however, neurogenesis (DCX) was significantly (p < 0.05) reduced by ramipril (Fig. 2). Ramipril (CTL-RAM) did not affect the density of CD68^+ ^activated microglia relative to CTL-SHAM. Relative to Control, progenitor proliferation and neurogenesis within the SGZ were significantly reduced following 10 Gy- (p < 0.001), and 15 Gy-WBI (p < 0.001), accompanied by a significant increase in CD68^+ ^activated microglia (p < 0.001). The decrease in neurogenesis was radiation-dose dependent (p < 0.01) (Fig. 2). Ramipril reduced the deleterious effects of radiation on progenitor proliferation (p < 0.01) and neurogenesis (p < 0.05) following 10 Gy-WBI, but was not effective following 15 Gy-WBI (Fig. 1). The mitigating effects of ramipril following 10 Gy-WBI were not accompanied by significant reductions in CD68^+ ^activated microglia counts (Fig. 3).

## Discussion

Our results add to a growing body of evidence suggesting that RAS inhibitors can successfully reduce radiation-induced late effects. As expected, based on previous reports, 10 Gy- and 15 Gy-WBI produced dose-dependent decreases in progenitor proliferation (Ki-67) and neurogenesis (DCX) accompanied by a dose-dependent increase in microglial activation within the dentate gyrus [5,9,19]. Our use of the term neurogenesis here refers specifically to the neurogenic potential of the SGZ, as evidenced by the induction of neuronal differentiation and the production of immature DCX^+ ^neurons within the SGZ microenvironment. Ramipril produced small but significant mitigating effects when administered following 10 Gy-WBI, but was not effective when administered following 15 Gy-WBI. The mitigating effects of ramipril were more pronounced for progenitor proliferation than they were for neurogenesis, suggesting that they may be mediated primarily by reducing radiation-induced apoptosis and/or mitotic catastrophes among progenitors and that the anti-inflammatory effects of ramipril may act less potently to mitigate radiation-induced disruption of neurogenic signaling within the SGZ. Ang II, acting primarily via AT2R, is a well established promoter of trauma-induced apoptosis in a variety of tissues, including brain, and administering AT2R antagonists has been shown to prevent Ang II-induced apoptosis [24-26]. Thus, inhibiting the production of Ang II with ramipril may similarly reduce apoptosis among neural progenitors within the SGZ following radiation injury. The rather limited mitigating effects produced by ramipril in this context may reflect that fact that the majority of radiation-induced apoptosis among progenitors within the SGZ occurs within 24 hours post-irradiation, preceding the initiation of ramipril therapy [35].

Cytostatic effects may also have contributed to the mitigation produced by ramipril in this context. In the Control group, ramipril significantly reduced neurogenesis without reducing progenitor proliferation within the SGZ, suggesting that the signaling required for neuronal (versus astrocytic) differentiation among these progenitors was selectively inhibited by ramipril. Cytostatic effects of ACEi and Ang II receptor antagonists are well established in vitro in a variety of normal and neoplastic cells [38], and also in vivo where they arrest fibroblast proliferation and regulate proliferation and migration of endothelial cells [39]. Mukuda et al. [40] recently reported that daily administration of the AT1R antagonist, losartan, maintained for 2 weeks suppresses running-enhanced increases in granule cell progenitor proliferation in the rat SGZ without affecting the basal proliferation rate. Ramipril's suppression of the basal rate of neurogenesis in the CTL-RAM group may reflect its different mechanism of action, since ACE inhibitors deprive both AT1R and AT2R of their substrate whereas losartan antagonizes only the AT1R. Alternatively, it may reflect the much longer duration of therapy and/or the relatively high dose of ramipril used in our study, which was approximately twice the standard clinical dose [41]. Both losartan and ramipril cross the blood-brain barrier and are thus able to influence the brain RAS system directly [42]. Ang II has been implicated as a promoter of vascular endothelial growth factor (VEGF) synthesis [41] and as a modulator of N-methyl-D-aspartate (NMDA)-glutamate receptor function [13,23]. Both of these processes are capable of acutely affecting the rate of neurogenesis within the SGZ and might therefore play a role in maintaining the basal rate of neurogenesis as well [40].

The mitigating effects of ramipril following 10 Gy-WBI were not accompanied by changes in the numbers of activated microglia, suggesting that the anti-inflammatory effects of ramipril may not have played a significant role. This observation lends additional support to an anti-apoptotic mechanism being primary in this regard. However, it is possible that ramipril produced anti-inflammatory/anti-oxidant effects by antagonizing the actions of IL-6, TNF-α, MCP-1, or other cytokines, without affecting the numbers of activated microglia [43]. It is also possible that radiation does not produce deleterious effects on progenitor proliferation and/or neuronal differentiation within the SGZ, but rather selectively suppresses expression of the two proteins we assayed as measures of these processes, i.e. Ki-67 and CD68, respectively. However, the results of numerous publications using a variety of assays for cellular proliferation and granule cell neurogenesis suggest this as an unlikely possibility [4-14]. Finally, the relevance of impaired granule cell neurogenesis to late delayed cognitive deficits has been called into question by recent reports indicating that such deficits can manifest in behavioral paradigms that are not conventionally viewed as hippocampal-dependent and at very long latencies (> 3 months) post-irradiation [44].

## Conclusions

Collectively our results add to the existing body of knowledge regarding the radiation dose-dependence of WBI effects on progenitor proliferation and neurogenesis within the SGZ, and establish that ramipril has the capacity to reduce the deleterious effects of WBI in this context. Though significant, the magnitude of these effects does not suggest the treatment regimen employed here as a promising therapeutic strategy in this regard. However, it is possible that these assays are not sufficiently inclusive and that the deleterious effects of irradiation on other processes pertinent to learning and memory are more potently mitigated by ramipril. More potent mitigating effects might also be achieved by administering ramipril at lower doses and/or for shorter durations, or in combination with other anti-apoptotic, anti-inflammatory or anti-oxidant therapies. Addressing these experimental issues will be the focus of future investigations.

## Competing interests

The authors declare that they have no competing interests.

## Authors' contributions

KAJ directed the project, performed cell counting, assisted with immunohistochemistry, and drafted the manuscript.

SLB co-directed the project and assisted with WBI.

JL administered WBI and experimental drugs, and performed perfusions and cell counting.

AK administered WBI and experimental drugs, and assisted with perfusions.

KL performed immunohistochemistry.

JHK directs the laboratory.

All authors read and approved the final manuscript.
